# Marital status, educational attainment, and suicide risk: a Norwegian register-based population study

**DOI:** 10.1186/s12963-021-00263-2

**Published:** 2021-07-12

**Authors:** Carine Øien-Ødegaard, Lars Johan Hauge, Anne Reneflot

**Affiliations:** Division of Mental and Physical Health, Norwegian Institute of Public Health, PO Box 222 Skøyen, 0213 Oslo, Norway

**Keywords:** Suicide risk, Risk factors, Educational attainment, Marital status, Sex, Register-data, Event history analysis

## Abstract

**Background:**

The presence and quality of social ties can influence suicide risk. In adulthood, the most common provider of such ties is one’s partner. As such, the link between marital status and suicide is well-documented, with lower suicide risk among married. However, the association between marital status and educational level suggest that marriage is becoming a privilege of the better educated. The relationship between educational attainment and suicide is somewhat ambiguous, although several studies argue that there is higher suicide risk among the less educated. This means that unmarried with low education may concurrently experience several risk factors for suicide. However, in many cases, these associations apply to men only, making it unclear whether they also refer to women. We aim to investigate the association between marital status, educational attainment, and suicide risk, and whether these associations differ across sexes.

**Methods:**

Our data consist of Norwegian residents aged 35–54, between 1975 and 2014. Using personal identification-numbers, we linked information from various registers, and applied event history analysis to estimate suicide risk, and predicted probabilities for comparisons across sexes.

**Results:**

Overall, associations across sexes are quite similar, thus contradicting several previous studies. Married men and women have lower suicide risk than unmarried, and divorced and separated have significant higher odds of suicide than never married, regardless of sex. Low educational attainment inflates the risk for both sexes, but high educational attainment is only associated with lower risk among men. Being a parent is associated with lower suicide risk for both sexes.

**Conclusions:**

Higher suicide risk among the divorced and separated points to suicide risk being associated with ceasing of social ties. This is the case for both sexes, and especially those with low educational attainment, which both healthcare professionals and people in general should be aware of in order to promote suicide prevention.

## Background

Suicide is a major public health issue, and about 600 lives are annually lost to suicide in Norway [1]. It is a complex phenomenon with multiple underlying causes, one of which can be the presence or absence of social ties and the quality of these. A better understanding of which characteristics inflate suicide risk is valuable in order to facilitate suicide prevention measures.

Durkheim investigated variations in social integration and its link to suicide mortality as early as 1897 [2], including the association between marital status and suicide rates. He argued that the reason for lower suicide risk among the married is that marriages provides stability, emotional support, and social integration, while separation, divorce, and widowhood rather promote social isolation [2]. The relationship between marital status and suicide risk has been reaffirmed by numerous studies [3–10]. However, the selection into marriage is an important element. Some have argued that there is an educational gradient into marriage [11, 12], suggesting that marriage is increasingly becoming a privilege of the better educated. In gender-egalitarian societies, like Norway, the educational effect on marriage is positive for both sexes [11]. In addition to a selection into marriage, there is seemingly an educational selection out of marriage as well [13], making the better-educated less likely to experience marriage dissolution. It is currently unestablished how the relationship between marital status and educational attainment together may affect suicide risk.

Educational inequalities are also observable in mortality rates. Educational differences in mortality, both absolute and relative, have increased since the 1960s [14, 15]. It is not clear if this also applies to suicide mortality, but several studies suggest this may be the case [14–20]. Durkheim’s theory on social integration and suicide can also be a part of the explanation of this relationship. The better educated have less chance of being unemployed [21], which is a known risk-factor for suicide, and when employed they often have more advanced occupations, which include more responsibilities, larger networks, and social ties [4, 22]. This sums up to a higher level of social integration.

In this paper, we aim to investigate the relationship between marital status, educational attainment, and suicide risk. Although it is well-known that marital status and educational attainment is strongly affiliated, it is unclear how this association in turn relates to suicide risk, and whether there are differences according to sex. This narrows the knowledge gap on suicide risk both because we investigate the interaction between marital status and educational attainment and obtain sex-specific results. Death by suicide is more common among men, and thus less is known about what increase and decrease the suicide risk among women. We will study this in a Norwegian setting by using large-scale register data covering the period 1975 to 2014. One of the main advantages of this study is the vast data source, providing reliability to the estimates for both sexes.

As Norway is one of the richest countries in the world, there are several features in the Norwegian society that seemingly could contribute to lower suicide risk. Firstly, there is approximately free primary, secondary, and tertiary education, which make the educational level of the population high compared to other countries [21, 23, 24]. Secondly, Norway also has a strong welfare state, providing a safety net for its inhabitants [25]. There are widely available primary healthcare services (PHC) throughout the country. In addition, the society is regarded gender-egalitarian [11], and women at large are financially independent of men. Despite all these structural characteristics, the suicide rate in Norway remains steady across years, and slightly above the average European rate [1, 26]. In sum, this adds up to making the Norwegian context appropriate to examine risk factors for suicide at an individual level.

The demography of the Norwegian population has undergone changes during the observation-period of this study. For instance, there has been a substantial increase in people with higher education, fewer are getting married [27], and the share of the population living alone has increased [28, 29]. This is also the case for the share of 45-year-olds who are childless [30]. The latter is particularly high among men, where 25% are still childless by the age of 45 [30]. Put together with the fact that men are overrepresented among suicide victims, and that the median age for suicide is 47 [1], we assume that new and updated information about the relationship between family situation and educational level can contribute to the knowledgebase for suicide preventive measures.

The data sources to be used are appropriate for studying the relationship between marital status, educational level, and suicide risk at an individual level. Norwegian registers cover the entire Norwegian population, thus reducing bias due to apostasy. They are updated regularly across decades, which allow for investigating rare phenomena like suicide, as well as providing information on time-varying aspects of the study population [31]. By linking several national registers, we have information on all the 4.073 suicides occurring in Norway in the age range 35 to 54, as well as demographic characteristics of the rest of the population of similar age. Examining the relationship between marital status, educational level, parenthood, and suicide has never been done in such a large-scale study population.

### Hypotheses

#### Marital status

The conclusions from studies investigating the relationship between marital status and suicide are quite similar. Being married represent both lower mortality risk in general [3, 8, 32] and lower suicide risk [4–7, 9, 33, 34], compared to widowhood, separation, divorce, and never being married. Two studies investigating both marital status and educational attainment, point to marital status as a more important factor for suicide risk than educational inequalities. Kravdal et al. find a growing mortality risk among the non-married, compared to married, and state that educational changes only explain up to 5% of the increase [8]. Lorant et al. have compared data from eight European countries and conclude that being married has a buffering effect against socioeconomic inequalities in suicide, although there are variations according to age [18]. Following these results, our first hypothesis is *(H1) being married is associated with lower suicide risk, also when taking account for educational attainment*.

#### Educational attainment

Following the theory regarding degree of social integration and risk of suicide, the assumption is that there is a negative relationship between educational attainment and suicide risk. Although this relationship is well investigated, the studies draw different conclusions. On the one hand, several studies do find higher suicide risk among less educated men [14–20], and except for two of these [17, 20], they also find a similar, but less consistent pattern for women. On the other hand, Lewis and Sloggett found no association between educational attainment and suicide risk [35]. Shah and Chatterjee [36] and Shah and Bhandarkar [37] found a curvilinear relationship between educational attainment and suicide risk, while Pompili et al. concluded with a high risk of suicide among the better educated [38]. Lusyne and Page also found higher risk of suicide among the better educated, but merely for women [34]. Most of these studies have in common the lack of control for marital status.

If in fact the better educated have a higher risk of suicide, but also a higher likelihood of getting married and thus a lower risk of suicide compared to the unmarried, we might expect to not find an association at all. It may be that these relationships cancel each other out. On the other hand, if there is an educational gradient into marriage and at the same time higher suicide risk among those with low educational attainment, this can mean accumulation of suicide risk among the less educated. Although the studies investigating educational attainment and suicide risk is somewhat inconclusive, studies regarding employment status and suicide find that unemployment is a large risk factor for suicide [39, 40]. We cannot argue that higher level of education equals employment, but it is two factors that are closely correlated. This association contributes to the expectation that higher educational attainment is associated with lower risk of suicide. Based on this, our second hypothesis is *(H2) there is a negative relationship between educational attainment and suicide risk, also when marital status is accounted for.*

#### Having children

Most children in Norway are born to married couples, although an increasing number are born to cohabitants [41]. Several studies find a substantial decrease in suicide risk among women with at least one child [33, 34, 42–44], but it is unclear if this also applies to men. One or more children adds another level of social integration to the marriage institution and may increase the level of social cohesiveness to the marital relationship. This can lead to an over-estimation of the effect of being married. However, the parenting bond is a social tie that is lasting, even in the case of marital disruption, and can thus be a protective factor against the elevated suicide risk of not being married. Our third hypothesis is *(H3) having at least one child is associated with lower suicide risk, in addition to the relationship described in (H1).*

#### Sex

Many of the studies regarding marital status and suicide risk find lower suicide risk among married men, while for women the association is weaker or uncertain [4, 6, 7, 9, 33]. When it comes to the relationship between educational attainment and suicide risk, the results are also ambiguous, with fewer and more contradictive results for women [14, 15, 17, 20, 45]. The relationship between having a child and suicide risk has opposite results, with few or weaker significant results for men [33, 34, 42–44]. It is difficult to know if there are different mechanisms in play according to sex, or if it is due to small sample samples. Some studies argue that there are different risk factors for suicide for men and women [33, 42, 43]. Still, several of the studies finding different results for men and women are cross-sectional and have few suicide cases. Especially when it comes to female suicide victims, it is difficult to identify significant risk factors due to few cases. As we have access to register data, covering the period 1975 to 2014, we assume we can identify the relationships, given that they in fact exist. Our fourth hypothesis is *(H4) the relationships described in H1-H3 apply to both sexes.*

## Methods

The aim of the study is to explore the association between marital status, educational attainment, and suicide risk. Marital status and educational attainment mutually affect each other, and it is currently unknown how this relationship influences suicide risk in Norway. We aim to investigate whether the association is different for men and women.

### Design and setting

The analyses are based on data covering the period 1975 to 2014 from the Norwegian Population Register, the Population Censuses, Statistics Norway’s Educational Registration System and the Norwegian Cause of Death Register. By means of unique personal identification numbers assigned to all Norwegian residents, it is possible to construct individual record linkages between different data sources. There are 668,332 men and 633,842 women, and 2871 male and 1202 female suicide victims. There are multiple records per person, one for each year they are under observation. In total, there are 25,351,586 person-years in the analyses.

The sample includes all residents of Norway born between 1940 and 1960 in the ages 35 to 54. The age range is based on several aspects. First of all, it includes the median age of suicide victims in Norway, which is 47 [1]. Second, the age of 35 is close to the mean age for first marriage, as well as the mean age for first separation and divorce is before 54 [46]. In addition, by the age of 35 most people have completed their education, so we limit the time-varying covariates to marital status, having a child and age. When individuals turn 55, emigrate or die, they are censored out.

We use discrete time event history analysis to investigate risk of suicide mortality. The data sources are updated annually, thus models utilizing discrete time events are appropriate. Our results stem from logit models using panel data with censoring, and thus the results are given in ORs. It is appropriate to use OR in this case, because we compare the odds of suicide in one group the odds of suicide in another group. Event history analysis is commonly used in epidemiology, and a good fit for estimating risk of suicide. We used Stata 16 to conduct the analyses. Odds ratios are relative results, and although fitting for investigating factors that impact the suicide risk, it is also a limitation. For instance, we cannot compare results across models (e.g., between sexes) and we cannot determine the absolute influences of these factors [47]. Thus, we have also estimated the marginal changes in predicted probability for suicide at the various educational levels and marital statuses. These results show the absolute change in predicted probability for suicide for different values and the covariates. This also provides the possibility to see how educational level and marital status pertain.

### Variables

The dependent variable is death by suicide during a year. This variable has the value “0” all the years prior to the suicide, and “1” in the year of death. The suicide victims are identified by the ICD-8 and ICD-9 codes E950–E958 and the ICD-10 codes X60–X84, Y870.

Marital status is categorized into five: never married, married, widowed, divorced, and separated. As of 2009, same sex couples are also included. We do not have information about couples that live together. The reference category is being married. Individuals without information regarding marital status were excluded from the analyses.

We grouped educational attainment in three levels: primary, secondary, and tertiary education. Primary education includes primary and middle school, secondary education refers to high school, while tertiary education means higher education. To be classified as either represents having completed this level of education. In the case of higher education, this means at least attained a bachelor’s degree. For the year(s) without information regarding educational level, the observations are dropped. The reference category for educational attainment is secondary education.

Having children is a dichotomous variable with the value 1 from the year of first child. If the transition to parenthood occurred prior to the observation period, the variable has the value 1 for all observations. Otherwise, the variable has the value 0.

To account for age, we have included a factor variable with 5-year age groups. They are “35–39”, “40–44”, “45–49”, and “50–54”. The third is the reference category, as it includes the median age for suicide in Norway.

The discrete time periods are a series of 1-year observations, and we included a time-variable counting the years under observation. For all the non-suicide victims, the maximum value for this variable is 20. The reference value is 10.

The models are separated by sex, as there may be different associations for men and women.

The Regional Committee for Medical and Health Research Ethics granted ethical approval for the main study.

## Results

The distribution of marital status and educational attainment amongst male and female suicide victims and the population controls are shown in Tables 1 and 2. Those who are currently married have a lower share of suicide victims. This holds for both men and women. For men, it is particularly never being married that has an overrepresentation of suicide victims, whereas for women it is being divorced. The share that is separated is three times as high among the suicide victims as for the population controls, for both men and women. Those with primary education are also overrepresented amongst the suicide victims. The share of men with tertiary education is considerably underrepresented amid the suicide victims, but this does not apply for the women with tertiary education. Women account for less than half of the suicides occurring between 1975 and 2014.

**Table 1:** Distribution of marital status among suicide victims and population controls. Share of suicide victims and person-years of the population controls within each category of marital status of Norwegian men and women aged 35 at start of 20 years follow-up during the period 1975–2014

Marital status	Men		Women	
	Suicide victims	Population controls	Suicide victims	Population controls
Never married	31% (880)	18%	17% (206)	11%
Married	38% (1085)	68%	42% (502)	71%
Widow/widower	1% (22)	0%	3% (39)	2%
Divorced	21% (617)	11%	29% (349)	13%
Separated	9% (267)	3%	9% (106)	3%
Total	100% (2871)	100%	100% (1202)	100%

**Table 2:** Distribution of educational attainment among suicide victims and population controls. Share of suicide victims and person-years of the population controls within each category of educational attainment of Norwegian men and women aged 35 at start of 20 years follow-up during the period 1975–2014

Educational attainment	Men		Women	
	Suicide victims	Population controls	Suicide victims	Population controls
Primary edu.	34% (982)	24%	34% (407)	28%
Secondary edu.	49% (1395)	50%	43% (511)	48%
Tertiary edu.	17% (494)	26%	24% (284)	25%
Total	100% (2871)	100%	100% (1202)	100%

### Event history analysis

Table 3 shows that the odds ratio for suicide mortality is significantly lower for the married, compared to the unmarried, regardless of sex. Being divorced or a widow/widower is associated with two to three times as high odds of suicide than the currently married. For men, never being married is also associated with over twice as high odds for suicide, than the married. However, as the confidence interval (CI) of being a widow/widower overlap with the other two, we cannot determine which status is most at risk. Although Table 1 shows that suicide victims are particularly overrepresented among the never married, Table 3 show that when controlled for education, child, and age, being divorced or separated is associated with a significantly higher suicide risk. The separated have over five times as high odds of death by suicide as the married, and for men the CI do not overlap with the CIs of any of the above-mentioned estimates. For women is there a slight overlap with the CI for the divorced, but the association is notably similar for both men and women.

**Table 3:** Odds ratio of suicide for men and women. Association between marital status, educational attainment, at least one child, and risk of suicide. Also controlled for age group and time

	Men		Women	
Variable	Odds ratio	95% CI	Odds ratio	95% CI
**Educational attainment** Ref: secondary education				
Primary education	1.27***	1.17–1.38	1.29***	1.13–1.47
Tertiary education	0.72***	0.65–0.80	1.03	0.89–1.19
**Marital status** Ref: married				
Never married	2.21***	1.98–2.48	1.75***	1.44–2.12
Widow/widower	2.78***	1.83–4.26	2.89***	2.08–4.01
Divorced	3.37***	3.05–3.73	3.60***	3.14–4.14
Separated	5.49***	4.80–6.28	5.03***	4.08–6.21
**At least one child**	0.63***	0.56–0.69	0.51***	0.43–0.61
_cons	0.00011***		0.00004***	
Prob > chi^2^	0.000		0.000	

*p < .05

**p < .01

***p < .001

Both men and women with primary education have higher odds of death by suicide than men and women with secondary education. Tertiary education is associated with lower odds for suicide, compared to the reference category for men, but not for women.

Having at least one child is associated with considerably lower odds for suicide, compared to the childless, for both men and women.

### Predicted probabilities

**Fig. 1:**
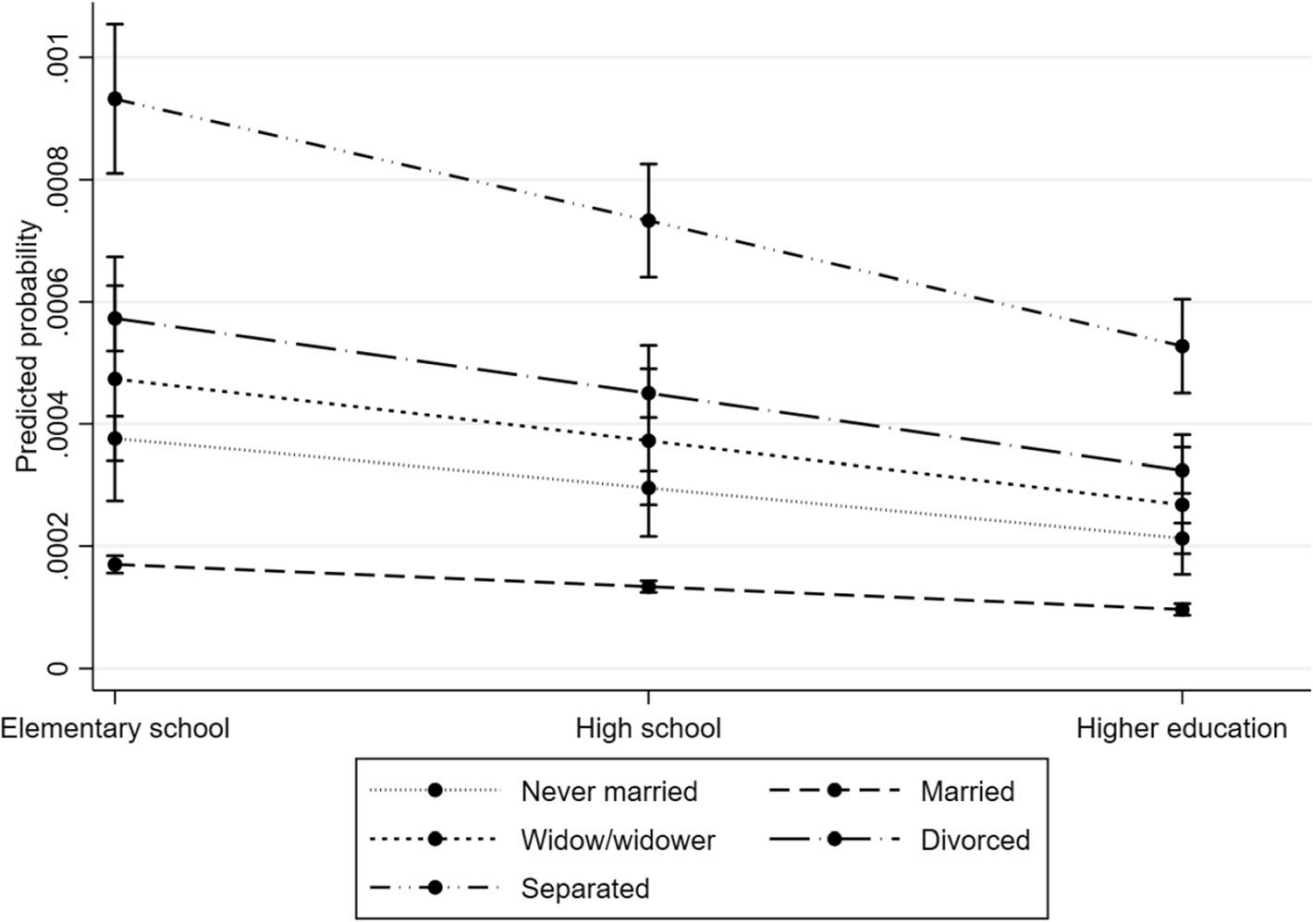
Predicted probability for suicide, men. Predicted probability for suicide within each category of marital status and educational attainment of Norwegian men aged 35 at start of 20 years follow-up during the period 1975-2014. Derived from the event history analysis, controlled for having a child age group and time

**Fig. 2:**
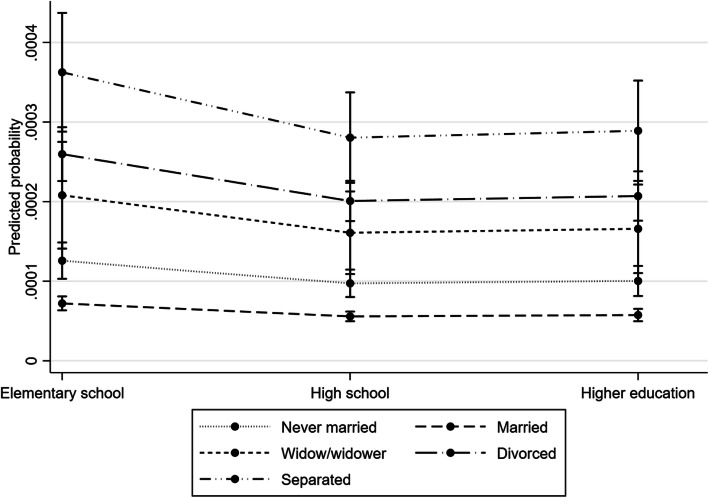
Predicted probability for suicide, women. Predicted probability for suicide within each category of marital status and educational attainment of Norwegian women aged 35 at start of 20 years follow-up during the period 1975-2014. Derived from the event history analysis, controlled for having a child age group and time

Figures 1 and 2 show the marginal changes in predicted probabilities for the various combinations of marital status and educational attainment. The predicted probabilities are low for everyone, but all the probabilities for men are about twice those of women. For men, educational attainment seem to have a linear relationship with probability for suicide, regardless of marital status: men with tertiary education have lower predicted probability of suicide than those with secondary education level, who again have lower predicted probability than those with primary education. There are striking variations according to marital status, but it is only being married or separated that are significantly different from the others. Although the predicted probabilities are at an overall low level, divorced men with primary education have as high as 0.0009 (almost one of 1000). That is significantly higher than separated men with tertiary education (about 0.0005) and much higher than married men with primary education (about 0.00015). For women, all the numbers are lower, but separated women with primary education also have the highest predicted probability for suicide (about 0.00036). Both sexes have significantly lower predicted probabilities among the married, regardless of educational level. The remaining categories of marital status are not significantly different from each other. For women, there is seemingly higher predicted probability of suicide amongst those with primary education, compared to those with secondary and tertiary education, but the estimates are not significantly different from each other.

## Discussion

This study indicates that there is a relationship between marital status, educational attainment, and suicide risk. Marital status accounts for the largest variation in the suicide risk. The married have consistently lower suicide risk than the other marital status groups. This is in line with previous research [5, 7–10], but some results have only been significantly for men [3, 4, 6]. Some of these did not account for educational level, and thus may the effect of marital status be inflated. In our analyses, educational attainment is included, thus our results lend support to our first hypothesis: *(H1) being married is associated with lower suicide risk, also when taking account for educational attainment*. These results are unambiguous across different models. Figures 1 and 2 show significant lower predicted probability for suicide among the married, which applies for all educational levels and for both sexes. Being married can seem to protect somewhat against educational inequalities in suicide, although the married with the lowest educational attainment have significantly higher odds for suicide than those with secondary and tertiary education. This substantiates the conclusion of a systematic review [18].

Being divorced or separated is associated with higher suicide risk than never being married, for both sexes and all educational levels (see Table 3). This can point to that marital dissolution is a substantial contributor to increased suicide risk. There is associated increased psychological distress in relation to union dissolution [9]. It may be that the dissolution of high-quality social ties is more of a risk factor for suicide than never having had such ties. However, some of the never married may be cohabitants, which can influence the estimation of suicide risk of this group. The separated have an odds ratio over five times as high as married do. This is in line with a former study, examining the association between being separated or divorced with suicide risk [9]. The predicted probability for suicide for the separated women at different educational levels is not significantly different from being divorced, but it is so for men (see Figs. 1 and 2). To our knowledge, no previous studies have found significant differences between divorced or separated women and never married women, accounted for educational attainment. As opposed to being divorced, separation is a status with a limited period that often involves conflicts, emotional distress, and major changes in living situation, everyday routines, and economic stability. It is thus likely that the especially elevated suicide risk we find among the separated may be an expression of the immediate reaction to the dissolution of the couple relationship, before it stabilizes at a slightly lower level. Still, this level is over three times as high as that of the married.

Our results indicate that low educational attainment is associated with higher suicide risk, and it seems as it pertains with marital status. This holds for both sexes. For men, we also find a lower suicide risk among the highest educated group, but having tertiary education do not seem to influence as much as having primary education. There are variations according to marital status for all levels of educational attainment. We do not find significant difference in suicide risk between women with secondary and tertiary education. In sum, our results give support to our second hypothesis *(H2) there is a negative relationship between educational attainment and suicide risk, also when marital status is accounted for.* These results are partly in line with previous studies. Several studies [14, 15, 18, 20] have found a negative relationship between educational attainment and suicide risk for men, and a less consistent pattern for women. Still, our results contradicts the curvilinear relationships found by Shah and Chatterjee [36], and Shah and Bhandarkar [37], and the positive relationship found by Pompili et al. [38]. Further, the abovementioned studies failed to account for marital status.

Kalmijn [11] found support for an educational gradient into marriage in gender-egalitarian societies, like Norway, and Murray [3] found lower mortality risk and protective effects of marriage itself. Our results point to an accumulation of suicide risk among the lower educated and not currently married. These results are even more severe as studies show that the better educated have far lower divorce risk than those with low educational attainment [13, 48]. This means that those with primary education, who are married, have higher risk of marriage dissolution and thus a rise in suicide risk.

Having at least one child is associated with lower odds ratio for suicide, for both men and women. This is an important finding. Most previous studies only find this relationship amongst women, but our results indicate that this relationship also is significant for men. There is a substantial difference in suicide risk (37%) between fathers and childless men. The parenting role can provide some of the social ties that disappear as the marriage dissolves, and from the results presented here it seem that these can be protective also for men. As these results are significant in a model containing both marital status and educational attainment, we conclude that we have support for our third hypothesis: *(H3) having at least one child is associated with lower suicide risk, in addition to the relationship described in (H1).*

Some studies point to similar relationships between the sexes when it comes to suicide risk and marital status [5, 8, 18, 32], educational attainment [14–16, 36–38], and having children [43]. Still, large parts of previous research on all three relationships find an association merely for one of the sexes [4, 6, 17, 20, 45]. Our results, however, point to comparable impacts. When it comes to suicide risk and marital status, the odds ratios in Table 3 are quite similar for men and women alike, although the standard errors for women are larger. Figures 1 and 2 show that although the pattern is parallel, the marginal change in predictive probability for different values of both marital status and educational attainment is much smaller for women. The married have significantly lower risk of suicide, which applies to both sexes. Low educational attainment also seems to have a similar association with suicide risk for both men and women. High educational attainment, on the other hand, has to some extent a protective impact on suicide risk for men, but not for women. Last, our results indicate that having a child is associated with substantially lower risk of suicide for both sexes. In sum, our results for the most part lend support to our fourth hypothesis: *(H4) the relationships described in H1-H3 apply to both sexes,* the only exception being lower suicide risk among those with high educational attainment.

Even though the associations and the odds ratios are similar for both sexes, Figs. 1 and 2 show that men have consistently higher predicted probability for death by suicide. This may be due to how men and women socialize. Women often have a higher number of close friends than men do, and thus higher degree of social integration [9] regardless of marital status or educational attainment, while men to a greater extent rely on their partner. Our data does not provide ground to state that this is the case, but it *can* be one of the reasons for why women have lower suicide rates than men. This can be a strengthening of the argument that risk factors for suicide should be investigated separately for men and women.

Durkheim argued that with a higher degree of social integration, meaning the strength and stability of social ties, the lower suicide risk a person has [2]. Two American cohort studies, aiming to measure the ranking of social integration, found that the higher grade, the lower the risk of suicide [39, 40]. Marriage is a source of a strong social integration and provides stability, a sense of meaning, and what Durkheim called a sense of cohesiveness [2, 6]. When the marriage ends, the ties are suddenly weakened, and perhaps absent all together, and thus the cohesion is disrupted. This results in increased risk of suicide [2]. However, marriage is not the sole source of social integration. It can help to have other forms of social ties. Those with higher education often have larger networks, and a stronger labor market-attachment [49], which can be of help in the case of marriage dissolution. Higher education has also been found to alleviate the risk of mortality in case of social isolation [50]. This corroborates the argument that those with higher educational attainment have greater chance of coping with not being married. Healthcare professionals, and perhaps most importantly the general practitioner (GP), should be aware of the elevated suicide risk of the non-married, especially at the time of separation, but also thereafter. They should also know that in co-occurrence with low educational attainment, the suicide risk is even greater.

Being unmarried and/or childless has a clear correlation with inflated suicide risk, regardless of educational attainment. As noted earlier, the marriage rate has declined during the observation period, whereas the share of the population living alone and the share of childless 45-year-olds are increasing. A recent narrative review found that being unmarried, living alone, and social isolation were some of the main predictors of suicidal behavior [10]. Our data cannot render information regarding feelings of loneliness or failure. However, the lack of close personal bonds seems to be a prominent risk factor for suicide, and a risk factor that is experienced among an increasing share of the population. Perhaps the possibility for outreach from (mental) health professionals in the case of separation, also in the absence of children, could be investigated.

In a systematic review of risk factors for suicide on population level, Li et al. argue that the overall importance of a risk factor is combined by the relative risk (RR) of said factor and its prevalence in the population [51]. Although some mental disorders have very high RR, their prevalence in the population is low. In contrast has socioeconomic deprivation relatively low RR, but high population prevalence. The authors then argue that suicide prevention strategies focusing on socioeconomic strata have the potential of similar population-level effects as strategies targeting the psychiatric risk factors [51]. Although this review does not mention marital status, being unmarried is also highly prevalent in the population, compared to severe mental disorders. Combining these results and insights implies that there is some unused potential for suicide prevention measures, for instance strategies targeting unmarried (men) with low educational level.

### Strengths and limitations

The main strength of this study is the high-quality register data-sources on which the analyses are conducted. We have had access to the entire Norwegian population, including all the suicides occurring within the observation period. This minimizes the challenges of attrition and selection bias. As the registers are updated annually, event history analyses are ideal to exploit much of the potential within the data-sources.

In this study, the person-years without information about educational attainment and/or marital status are excluded. The share of informants without information regarding education is 10%, and the same is the share of observations. The equivalent shares without data regarding marital status are 0.67% and 2%. Immigrants are highly overrepresented in these groups. This obviously adds bias to the results, and we cannot conclude that our results also apply for the immigrant population at large.

Our results are limited to the age groups included. A meta study of sociodemographic risk factors for suicide found that the rarer the factor, the greater the impact [52]. The risk factors found in this study may very well have different implication for the younger and the elderly.

The registers do not contain information regarding cohabitation. It is common in Norway to live together for several years prior to marriage, and it is estimated that there are 600,000 cohabitates in Norway [53]. About 25% of children in Norway lives with their cohabiting parents [54]. It is possible then, that some in other categories than being married, in fact are in marriage-like relationships. This may contribute to an underestimation of suicide risk in all the unmarried categories. From 2009, married same sex couples are included. Prior to this year, same sex-couples are also grouped within the “never married” category.

The observation period covers several decades, and the risk related to the various marital status categories may have changed over time. Tests were executed with a period-interaction term and a period-control variable, which did not give ground for a period effect. Still, the estimates presented in the results section are averaged across the timespan and may not reflect the yearly suicide risk.

### Future research

From the results presented here, some new research questions arise. Firstly, future research could investigate the aspect of timing. We find that the separated have particularly high suicide risk, also in comparison with the divorced. The widow/widower-category includes all living in widowhood, regardless of years since the spouses’ passing. However, it may be that the recently widowed have higher suicide risk, due to the recentness of the union dissolvent.

Our data sources lack the opportunity to investigate cohabitation. A large part of the relationships, especially among young people, are cohabitant couples and it would be interesting to investigate if this provides the same protective factor as marriage.

Our study has parenthood as a dichotomous variable. Future research could investigate this relationship further. For instance, does number of children, age of child(ren), living situation (fulltime/part time/nothing), or presence of stepchildren probably impact the relationship.

We use educational attainment as a measurement for socioeconomic status, but a more composed variable could be advantageous. For instance, would income, occupation, and/or spouses’ income add value to the results?

Studies regarding PHC-usage, both in the population in general and among suicide victims, show that women are more frequent users of the healthcare system [55]. Future research could investigate if there are inequalities in health care usage according to educational level and/or marital status. This would extend the knowledge of what triggers the results presented in this study.

## Conclusion

In this study, we have investigated the relationship between marital status, educational attainment, and suicide risk. Our results point to that high degree of social integration is associated with lower suicide risk. The presence or absence of close personal ties is especially important. Having a child is associated with lower suicide risk for men as well as for women, and union dissolution inflates the suicide risk considerably for both sexes. For men, it is seemingly an accumulation of suicide risk of the unmarried with lower education. Men with primary education have the highest suicide risk within any category of marital status. Separated men with primary education also have the highest predicted probability for death by suicide. Likewise, the men with tertiary education have the lowest suicide risk within each category of marital status. The results for women are similar to those of men, except that we do not find any difference in suicide risk between women with secondary and tertiary education. All categories of not being currently married are associated with higher odds of suicide for women, and we find particularly high odds among the divorced and separated. The findings presented here thus suggest that the consequence of marriage dissolution is an elevated suicide risk for both sexes, and those with primary education may be especially exposed. The possibility of extra follow-up at the time of marriage dissolution, by the GP or others in the health care system, should be investigated. In addition, suicide prevention strategies focusing on strengthening the presence of social ties should be explored. This is particularly important for those experiencing other known risk factors for suicide.

## Data Availability

The data that support the findings of this study are available from Cause of Death Registry and Statistics Norway, but restrictions due to privacy apply to the availability of these data, which were used under license for the current study, and so are not publicly available.

## Abbreviations

PHC: Primary health care
ICD: International Classification of Diseases
CI: Confidence interval
GP: General practitioner
RR: Relative risk
